# Family Tree Mapping of Genetic and Phenotypic Codes of Hereditary Angioedema

**DOI:** 10.3390/medicina62091635

**Published:** 2026-08-26

**Authors:** İlke Baş, Ela Gökmen, Türkan Dizdar Canbaz, Ecem Ay, Ragıp Fatih Kural, Eda Aslan, Ümitcan Ateş, Kutay Kırdök, Asli Kilavuz, Mustafa Eren Kaçmaz, Ayça Aykut, Elif Ayaz, Füsun Özyaman, Figen Gülen, Handan Duman Şenol

**Affiliations:** 1 Department of Pediatric Allergy and Immunology, Faculty of Medicine, Ege University, İzmir 35100, Türkiye; 2 Izmir American Collegiate Institute, İzmir 35290, Türkiye; 3 Department of Allergy and Immunology, Faculty of Medicine, Ege University, İzmir 35100, Türkiye; turkan88dizdar@gmail.com (T.D.C.); edaarslan_91@hotmail.com (E.A.);; 4 Department of Internal Medicine, Faculty of Medicine, Ege University, İzmir 35100, Türkiye; asli.kilavuz@ege.edu.tr; 5 Department of Medical Genetics, Faculty of Medicine, Ege University, İzmir 35100, Türkiye

**Keywords:** hereditary angioedema, *SERPING1*, C1 inhibitor

## Abstract

*Background and Objectives*: Hereditary angioedema due to C1 inhibitor deficiency (HAE-C1INH) is a rare autosomal dominant disorder caused by mutations in the *SERPING1* gene and is characterized by considerable clinical variability among affected individuals. Although genotype–phenotype correlations have been reported, data from populations with high consanguinity rates remain limited. Therefore, this study aimed to construct pedigrees of HAE-C1INH families harboring *SERPING1* mutations and to investigate whether mutation type is associated with phenotypic characteristics, particularly age at disease onset and C1 inhibitor functional activity. *Materials and Methods*: Eleven HAE-C1INH probands and their families were followed up at the Allergy and Immunology Department of Internal Medicine, Ege University Faculty of Medicine. Of the 295 family members identified through pedigree construction, 109 had symptoms compatible with HAE-C1INH, and 53 of these symptomatic individuals had both a confirmed *SERPING1* genetic result and an available C1-INH functional measurement. Pedigrees were created using the Progeny Pedigree Drawing Software (Progeny Genetics LLC, Delray Beach, FL, USA). Demographic data, disease characteristics, C1-INH function levels, and *SERPING1* mutation types were obtained from patient files. *Results*: A total of 295 individuals (151 males and 144 females) from 11 HAE-C1INH families were identified through pedigree construction. The analytical cohort comprised 53 symptomatic individuals, of whom 7 (13.2%) were homozygous for the variant. Among heterozygous patients (*n* = 46), 27 (58.7%) had missense mutations, 7 (15.2%) had nonsense mutations, 7 (15.2%) had large deletions, and 5 (10.9%) had frameshift deletion mutations. Patients carrying missense mutations had significantly higher median C1-INH functional activity (28.5%) than those with other mutation types (14.1%) (*p* = 0.026), and the median age of disease onset was significantly later in the missense group (20 years) than in the non-missense group (8 years) (*p* < 0.001). Annual HAE attack frequency was also lower in the missense group (median, 12 vs. 48 attacks per year; *p* = 0.009). *Conclusions*: Exploratory individual-level analyses showed higher residual C1-INH functional activity and later symptom onset among individuals carrying missense *SERPING1* variants than among those carrying pooled non-missense loss-of-function variants. However, familial clustering and the small number of independent families limit the strength and generalizability of these findings.

## 1. Introduction

Hereditary angioedema due to C1 inhibitor deficiency (HAE-C1INH) is a rare autosomal dominant disorder with an estimated prevalence of approximately 1:10,000–1:50,000. It is characterized by unpredictable, recurrent episodes of non-pruritic swelling affecting the skin, mucosal tissues, and internal organs [1,2,3]. It occurs due to dysfunction or deficiency of the C1 inhibitor (C1-INH), a serine protease inhibitor encoded by the *SERPING1* gene located on chromosome 11q12–q13.1, which comprises eight exons [4,5]. Diagnosis is established by demonstrating low C1-INH function (and/or low C1-INH antigen level) and confirmed by detecting pathogenic variants in the *SERPING1* gene through genetic analysis [4,6]. Although HAE-C1INH is a genetic disease, symptoms do not appear immediately after birth; the median age of symptom onset is approximately 10 years, but onset can range from early childhood to over 60 years of age [1,7]. Earlier disease onset and lower C1-INH function levels have been associated with a more severe disease course [7,8].

Ponard et al. [5] catalogued 748 different *SERPING1* gene variants, of which 36.2% were short deletions, duplications, and indels causing frameshifts, 32.1% were missense variants, 13.6% were splicing defects, 9% were nonsense variants, and 8.2% were large deletions and duplications. Over 500 of these have been registered in the Human Gene Mutation Database (HGMD) as disease-causing mutations. Several studies have investigated the genotype–phenotype correlations in HAE-C1INH. Bors et al. [9] first demonstrated that patients with missense mutations had less severe clinical manifestations than those with other types of mutations. A systematic review by Loli-Ausejo et al. [10] identified 12 published studies on this topic and found that missense mutations were consistently associated with delayed disease onset and lower severity scores. Similarly, a Czech national cohort study by Grombirikova et al. [11] demonstrated a significantly delayed disease onset in patients with missense variants compared to that in patients with null variants. Mete Gökmen et al. [8] examined the relationship between *SERPING1* deletions and C1-INH function levels, showing that lower C1-INH function predicted disease severity.

However, data on genotype–phenotype correlations in populations with high rates of consanguineous marriages remain limited. Consanguinity increases the likelihood of homozygosity for *SERPING1* variants, which has been reported in only a few families worldwide [12,13,14]. Turkey has one of the highest rates of consanguineous marriage in the world, estimated at 20–25% [15], making it an important population for studying the effects of homozygosity on the HAE-C1INH phenotype.

Despite growing evidence supporting genotype–phenotype associations in HAE-C1INH, important gaps remain regarding pedigree-based analyses in populations with high rates of consanguinity and the phenotypic consequences of homozygous *SERPING1* variants. In this study, we constructed detailed pedigrees of 11 Turkish families with HAE-C1INH and investigated whether *SERPING1* mutation type was associated with residual C1-INH function and age at disease onset. We further evaluated the clinical and biochemical impact of homozygosity in consanguineous families. We therefore hypothesized that *SERPING1* mutation class influences both biochemical severity and clinical phenotype and that pedigree analysis may improve genotype-based risk stratification in HAE-C1INH.

## 2. Materials and Methods

### 2.1. Study Design

This retrospective, single-center, pedigree-based cross-sectional study included genetically confirmed probands with hereditary angioedema due to C1 inhibitor deficiency (HAE-C1INH) and their family members who were followed at the Department of Allergy and Immunology, Department of Internal Medicine, Ege University Faculty of Medicine, İzmir, Türkiye.

### 2.2. Study Population

A total of 11 genetically confirmed HAE-C1INH probands and their families were included in the study. Demographic characteristics, age at disease onset, genetic findings, and C1-INH functional activity were obtained retrospectively from medical records.

For genotype–phenotype analyses, only individuals with genetically confirmed *SERPING1* variants and available C1-INH functional measurements were included. Family members without genetic confirmation or with incomplete biochemical data were excluded from comparative analyses but were retained for pedigree construction.

### 2.3. Pedigree Construction

Pedigrees were generated using Progeny Pedigree Drawing Software (Progeny Genetics LLC, Aliso Viejo, CA, USA; https://progenygenetics.com/clinical/pedigree/; accessed on 21 August 2026). Family members were identified through detailed pedigree assessment, and clinical information was integrated with available genetic and biochemical data to evaluate intrafamilial genotype–phenotype relationships.

To ensure consistency between the pedigree symbols and the analytical dataset, the pedigrees were cross-checked against the available clinical, genetic, and biochemical records. Supplementary Table S1 presents individual-level data for all 53 symptomatic participants included in the genotype–phenotype analysis. Complete and verifiable individual-level genetic and biochemical data were not available for several other relatives depicted in the pedigrees because they had been identified retrospectively through family-history assessment and had not undergone complete genetic and biochemical evaluation. These individuals are therefore represented using family-specific summary counts rather than individual-level clinical data.

### 2.4. Genetic Analysis

Genetic testing was performed using Sanger sequencing of all coding exons and exon–intron boundaries of the *SERPING1* gene. Multiplex ligation-dependent probe amplification (MLPA) was additionally performed to detect large deletions and duplications. Identified variants were classified according to the recommendations of the American College of Medical Genetics and Genomics and the Association for Molecular Pathology (ACMG/AMP) guidelines [16]. Variant types were categorized as missense, nonsense, frameshift deletion, or large deletion involving one or more exons. Genetic testing indications and interpretation followed the international consensus recommendations for the genetic diagnosis and management of hereditary angioedema [6].

### 2.5. C1-INH Functional Assay and Clinical Assessment

Functional C1 inhibitor activity was measured using a chromogenic assay according to the manufacturer’s recommendations. C1-INH functional activity, *SERPING1* variant type, age at symptom onset, number of HAE attacks per year, history of laryngeal attacks, HAE-related hospitalization, and long-term prophylaxis use were retrospectively extracted from the medical records. The number of HAE attacks per year was defined as the documented number of attacks during the 12 months preceding the most recent clinical assessment. Bygum and Freiberger severity scores were recorded when available from routine clinical assessments. Missing severity scores were not retrospectively calculated or imputed. These clinical, genetic, and biochemical variables were used for genotype–phenotype analyses, and individual-level data are provided in Supplementary Table S1.

### 2.6. Statistical Analysis

Statistical analyses were performed using IBM SPSS Statistics for Windows, version 25.0 (IBM Corp., Armonk, NY, USA). Categorical variables are presented as numbers and percentages, and continuous variables as medians and ranges. Distributional characteristics and sample size were considered when selecting statistical tests; accordingly, non-parametric methods were used for group comparisons.

The primary exploratory analysis was restricted to symptomatic heterozygous individuals. Because the nonsense, frameshift-deletion, and large-deletion subgroups were small, they were pooled into a non-missense loss-of-function group and compared with the missense group using the Mann–Whitney U test. The individual non-missense subgroups were summarized descriptively. Secondary continuous outcomes were compared using the Mann–Whitney U test and categorical outcomes using Fisher’s exact test. Severity-score analyses were based on available cases without imputation. Homozygous individuals were evaluated separately using descriptive statistics only.

Because participants were clustered within 11 families and variant class was constant within each family, the observations were not fully independent. The small number of families and the sparse, structurally unbalanced data precluded reliable family-adjusted or multivariable modelling. Therefore, all individual-level comparisons were considered exploratory and unadjusted, and *p*-values were interpreted cautiously. All tests were two-sided, and *p* < 0.05 was considered statistically significant.

## 3. Results

Of the 295 individuals represented in the pedigrees, 109, including the 11 probands, had symptoms compatible with HAE-C1INH. Of these, 53 had both a confirmed *SERPING1* genetic result and an available C1-INH functional measurement and were included in the genotype–phenotype analysis; symptomatic individuals lacking either or both results were excluded from the comparative analyses. Among the 53 included individuals, 46 (86.8%) were heterozygous and 7 (13.2%) were homozygous for the familial variant. All seven homozygous individuals carried missense variants (Table 1).

The other family members represented in the pedigrees either had incomplete clinical, genetic, or biochemical data, had been confirmed as non-carriers, or had not undergone genetic testing.

**Figure 1:**
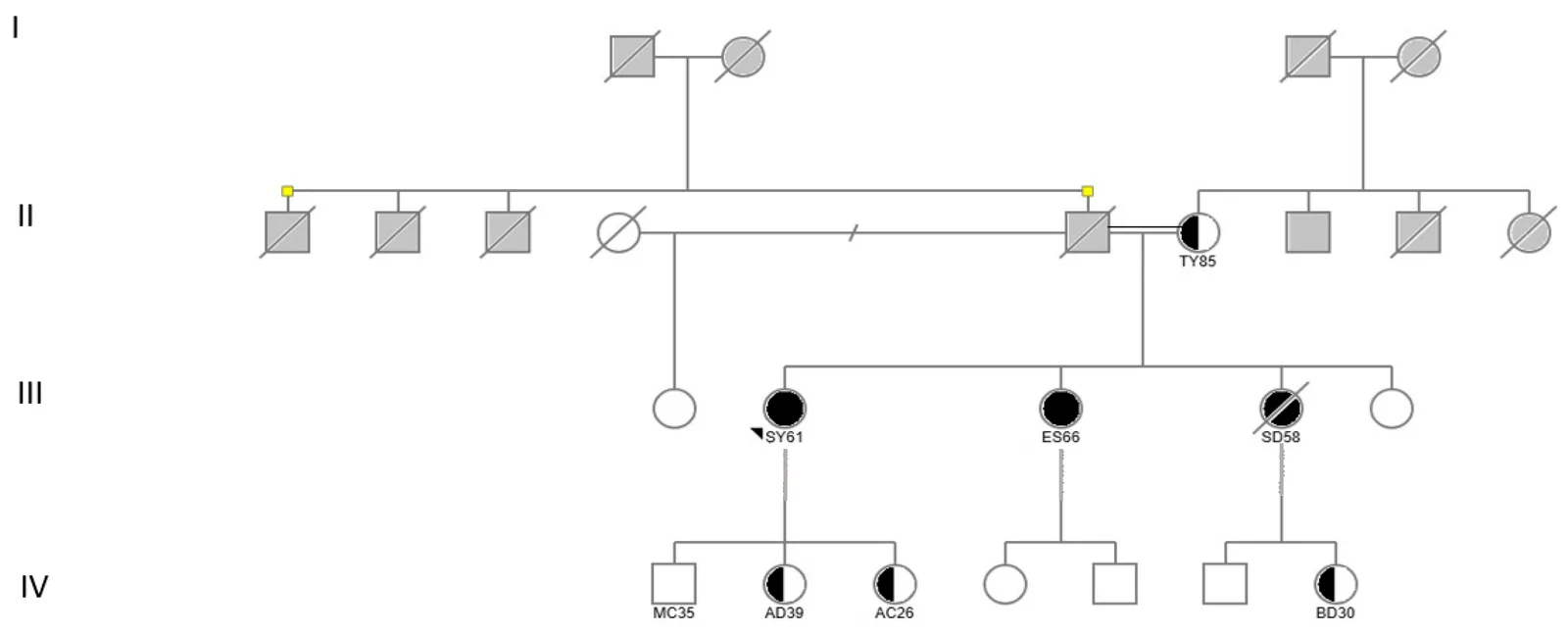
Representative pedigree of Family G illustrating parental consanguinity and individuals carrying the familial SERPING1 variant. Squares indicate males, circles indicate females, and diagonal lines indicate deceased individuals. Filled symbols represent homozygous individuals, half-filled symbols represent heterozygous individuals, open symbols represent individuals in whom the familial variant was not detected, and grey symbols represent individuals with a family history consistent with HAE-C1INH who did not undergo genetic testing. The double horizontal line indicates consanguinity, and the arrow identifies the proband. Roman numerals I–IV indicate successive generations.

A representative pedigree illustrating the coexistence of heterozygous and homozygous individuals is presented in Figure 1. Complete pedigrees of all 11 families are provided in Supplementary Figure S1.

Consanguineous marriages were present in families with homozygous individuals (Families G, I, and J; pedigrees in Supplementary Figure S1). In these families, certain heterozygous carriers of missense variants remained clinically asymptomatic at the time of assessment. Homozygous individuals showed heterogeneous clinical and biochemical profiles, with earlier symptom onset and lower C1-INH functional activity observed in some, but not all, families. Although these observations may be compatible with a gene-dosage effect in certain families, the small number of homozygous individuals precludes definitive conclusions. Notably, the heterozygous carriers in these families had C1-INH functional activity below the normal range, indicating biochemical expression of the familial variant in the heterozygous state. This pattern is consistent with autosomal dominant inheritance with variable expressivity. The pedigrees and the analytical dataset were reconciled at the individual level. Individual-level data for participants included in the primary analysis and family-level summaries for the remaining pedigree members are provided in Supplementary Table S1.

Among the 46 symptomatic heterozygous individuals, 27 (58.7%) carried missense variants and 19 (41.3%) carried pooled non-missense loss-of-function variants, comprising 7 nonsense variants, 5 frameshift deletions, and 7 large deletions. Age at symptom onset was available for all 46 individuals. Because of their small sample sizes, the nonsense, frameshift deletion, and large-deletion subgroups are presented descriptively only; no inferential comparison was performed across the four individual variant categories (Table 2).

**Figure 2:**
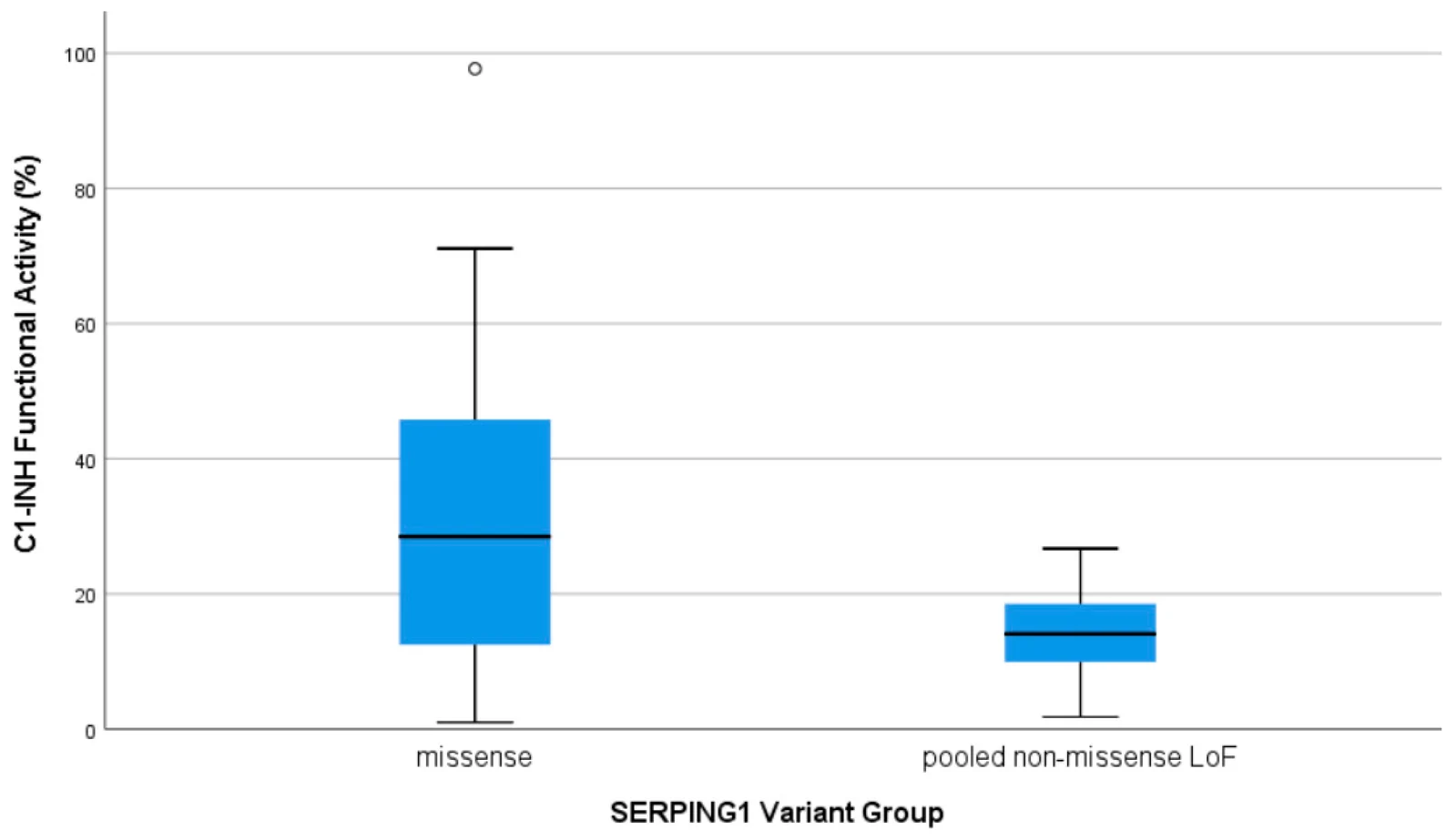
Distribution of C1-INH functional activity among symptomatic heterozygous individuals carrying missense and pooled non-missense loss-of-function variants. Boxplots show the median, interquartile range, and whiskers. The open circle indicates an outlier, defined as a value more than 1.5 times the interquartile range beyond the box.

In the exploratory two-group analysis, patients with missense variants had higher median C1-INH functional activity than those carrying pooled non-missense loss-of-function variants (28.5% [range, 1–97.7%] vs. 14.1% [range, 1.8–26.7%]; *p* = 0.026; Figure 2). Median age at symptom onset was also later in the missense group (20 years [range, 7–70 years] vs. 8 years [range, 4–25 years]; *p* < 0.001). These 46 individuals were clustered within 11 families, comprising 7 families carrying missense variants and 4 families carrying non-missense loss-of-function variants.

Additional clinical characteristics and severity measures are presented in Table 3. The annual number of HAE attacks was significantly lower in the missense group than in the pooled non-missense loss-of-function group (median, 12 [range, 1–227] vs. 48 [range, 4–225] attacks per year; *p* = 0.009). No significant between-group differences were observed in laryngeal attack history, HAE-related hospitalization, or long-term prophylaxis use (all *p* > 0.05). Bygum and Freiberger severity scores were available for 31 individuals: 20 of 27 in the missense group and 11 of 19 in the pooled non-missense loss-of-function group. Neither score differed significantly between the groups (*p* = 0.526 and *p* = 0.95, respectively). These secondary analyses were considered exploratory because of incomplete data, familial clustering, and multiple comparisons.

The individual clinical, genetic, and biochemical characteristics of the seven homozygous individuals from Families G, I, and J are presented in Table 4. Considerable clinical and biochemical heterogeneity was observed across families. Homozygous individuals in Families G and J showed earlier symptom onset and lower C1-INH functional activity than those in Family I. Because of the small number of homozygous individuals, their clustering within three families, and the absence of an independent comparison group, these observations were interpreted descriptively, and no formal inferential analysis was performed.

## 4. Discussion

The present study provides pedigree-based evidence that SERPING1 variant type may be associated with phenotypic heterogeneity in HAE-C1INH. Three principal findings emerged. First, in exploratory individual-level analyses, heterozygous individuals carrying missense variants showed higher residual C1-INH functional activity and later symptom onset than those carrying pooled non-missense variants. Second, homozygous individuals from consanguineous families showed heterogeneous clinical and biochemical profiles, suggesting that the phenotypic consequences of homozygosity may vary according to the specific variant and familial context. Third, pedigree analysis illustrated substantial intrafamilial phenotypic variability despite shared pathogenic variants.

In addition to higher residual C1-INH functional activity and later symptom onset, individuals carrying missense variants had a significantly lower annual HAE attack frequency than those carrying pooled non-missense loss-of-function variants. However, no significant differences were observed in laryngeal attack history, HAE-related hospitalization, long-term prophylaxis use, or the available Bygum and Freiberger severity scores. Therefore, the findings suggest a lower attack burden in the missense group but do not establish a uniformly milder clinical phenotype.

An association between missense mutations and milder disease expression was first reported by Bors et al. [9] in a Hungarian cohort of 106 patients, who showed that missense mutation carriers had a lower annual attack frequency, less use of subcutaneous medication, and fewer abdominal attacks. A subsequent overview of *SERPING1* variants in Hungarian patients by Szabó et al. [17] further characterized the spectrum of variants in this population. Our findings corroborate the observation of a later disease onset in the missense group. Similar genotype–phenotype patterns have been observed in other European cohorts, including studies from southeastern European populations [18] and a Macedonian cohort by Grivčeva-Panovska et al. [19], in which mutations with a clear effect on C1-INH function were associated with a more severe disease phenotype. Loli-Ausejo et al. [10], in a systematic review and a Spanish cohort study of 88 adult HAE-C1INH patients, identified twelve published manuscripts on genotype/phenotype correlations and confirmed that missense mutations in the *SERPING1* gene were consistently associated with delayed disease onset and lower severity scores across studies. Interestingly, their own cohort data showed only minimal differences in clinical phenotypes among the mutation classes, with class III mutations (including missense variants affecting the reactive center loop) tending toward a more severe phenotype, suggesting that the specific location of the missense variant within the protein may further modulate clinical expression.

Drouet et al. [12] comprehensively characterized the relationship between *SERPING1* variants and C1-INH function and showed that both haploinsufficiency and dominant-negative mechanisms contribute to disease severity. Variants affecting structurally important regions of the serpin molecule may retain partial protein production while impairing function, thereby explaining the broad spectrum of residual C1-INH activity observed among patients with missense variants in our cohort. This heterogeneity is also evident in the broad distribution of C1-INH functional activity within the missense group shown in Figure 2.

More recent national cohort studies have further strengthened the evidence for genotype–phenotype correlations. Grombirikova et al. [11], in a systematic screening of 207 Czech patients from 85 families, demonstrated a significant association between HAE type 1 missense variants and delayed disease onset compared with null variants (*p* = 0.023). They also identified a significant correlation between the *SERPING1* variant c.-21T>C in the trans position and attack frequency, disease onset, and clinical severity score, suggesting that regulatory variants may function as disease modifiers. This finding aligns with the earlier work by Speletas et al. [20], who demonstrated that the F12-46C/T polymorphism could modify the clinical phenotype of HAE, with the CC genotype associated with an earlier disease onset. The Slovak national cohort study by Markocsy et al. [21], which analyzed 132 patients and identified 22 unique causal variants, including 12 novel ones, further expanded the known variant spectrum and confirmed that comprehensive screening strategies are essential for improving diagnosis and understanding of genotype–phenotype relationships.

At the molecular level, Haslund et al. [22] elucidated the dominant-negative mechanism through which certain *SERPING1* missense mutations cause intracellular retention and aggregation of the C1-INH protein in the endoplasmic reticulum. They demonstrated that the mutated C1-INH protein can interact with and trap wild-type C1-INH in intracellular aggregates, reducing the secretion of functional proteins. Critically, the severity of this aggregate formation varied among different mutations, providing a structural explanation for the different levels of residual C1-INH function produced by different missense mutations. More recently, Jiang et al. [23], in a 2024 study, discovered a novel SERPING1 variant (c.708T>G; p.Phe236Leu) that also caused intracellular C1-INH accumulation and triggered downstream Ca^2+^ overload, mitochondrial damage, and apoptosis, suggesting additional pathogenic mechanisms beyond simple protein retention. Together, these mechanistic data provide a biologically plausible explanation for our findings by demonstrating how different missense variants can preserve variable levels of residual C1-INH activity, whereas null variants generally abolish functional protein production.

Annual HAE attack frequency was significantly lower among individuals carrying missense variants than among those carrying pooled non-missense loss-of-function variants. This finding is directionally consistent with the later symptom onset and higher residual C1-INH functional activity observed in the missense group. Nevertheless, attack frequency may be influenced by age, treatment exposure, access to care, recall accuracy, and individual or familial disease-modifying factors. Moreover, the analysis was unadjusted for familial clustering and multiple secondary comparisons. Therefore, this association should be interpreted as exploratory and requires confirmation in larger, independent cohorts.

One of the distinctive findings of this study was the identification of seven homozygous individuals from three consanguineous families (Families G, I, and J). These individuals showed heterogeneous clinical and biochemical characteristics. Homozygous individuals in Families G and J showed earlier symptom onset and lower C1-INH functional activity, whereas those in Family I had comparatively later symptom onset and higher residual functional activity. These observations suggest that the phenotypic consequences of homozygosity may depend on the specific variant and familial context. Mete Gökmen et al. [14], in a 2020 study in Turkey, described two families with novel homozygous SERPING1 variants, including a homozygous c.1202T>C (p.Ile401Thr) missense mutation in exon 7 associated with delayed disease onset and milder clinical course, and a homozygous c.1379C>T (p.Ser460Phe) missense mutation in exon 8 associated with severe disease and extremely low C1-INH function. These contrasting phenotypes highlight that the specific variant, not merely the zygosity status, determines the clinical outcome in homozygous patients.

However, we must emphasize that the inheritance pattern in our consanguineous families is best described as autosomal dominant with variable expressivity and not autosomal recessive. The heterozygous carriers in these families had low C1-INH function levels below the normal range, demonstrating that the biochemical defect was expressed in the heterozygous state. This is fundamentally different from true autosomal recessive inheritance, in which heterozygous carriers would exhibit normal C1-INH levels and function, as described for the p.Val322Met variant by Maia et al. [13]. The “asymptomatic” heterozygous carriers identified in these families may reflect the variable and age-dependent clinical expression of HAE-C1INH. Although they had not developed clinical symptoms at the time of assessment, they already exhibited biochemical evidence of C1-INH deficiency. Drouet et al. [12] reported that truly recessive *SERPING1* variants represent approximately 5.8% of all pathogenic variants and are typically classified as benign or of uncertain significance by prediction tools, which is not the case for the variants identified in our families. Therefore, the observations in our consanguineous families are consistent with autosomal dominant inheritance with variable expressivity. Although homozygosity may contribute to greater biochemical or clinical expression in some families, the limited number of homozygous individuals and the observed interfamily heterogeneity preclude definitive conclusions regarding a uniform gene-dosage effect.

Our previous study [8] demonstrated that deletions in *SERPING1* led to lower C1-INH function and that lower C1-INH function could predict disease severity, establishing the importance of C1-INH function level as a phenotypic marker of HAE. The present study extends these observations by showing that mutation type is associated with both C1-INH functional activity and age at symptom onset, providing a potential genetic basis for the observed biochemical–clinical relationship.

### Limitations

This study has several limitations. First, the small non-missense subgroups (*n* = 5–7) precluded reliable four-group comparisons. These variants were therefore pooled into a non-missense loss-of-function group, and the two-group analyses should be considered exploratory. Second, participants were clustered within 11 families and were not fully independent. Familial clustering may have inflated statistical significance, whereas the small number of families precluded reliable family-adjusted or multivariable modelling; residual confounding therefore cannot be excluded. Third, the retrospective design and incomplete data may have introduced selection bias. Moreover, only 53 of the 109 symptomatic individuals had both a confirmed *SERPING1* result and an available C1-INH functional measurement. Individuals with complete genetic and biochemical evaluations may have differed systematically from those with incomplete assessments, introducing potential selection bias and limiting the representativeness of the analytical cohort. Bygum and Freiberger severity scores were available for only 31 of the 46 symptomatic heterozygous individuals and may not have been missing at random, limiting comparisons of clinical severity between variant groups. In addition, the significant difference in annual attack frequency arose from a secondary, unadjusted analysis and was not corrected for familial clustering or multiple comparisons; therefore, it should be interpreted cautiously. Finally, age at symptom onset was available for all symptomatic heterozygous individuals included in the revised analysis. However, asymptomatic carriers could not be included in this analysis because they had no definable age at symptom onset. Restricting the analysis to symptomatic individuals may have introduced additional selection bias and increased the apparent difference between the variant groups. Therefore, the findings should not be interpreted as an unbiased estimate of age-dependent penetrance, and prospective follow-up of asymptomatic carriers is required.

Overall, these findings suggest that mutation class should be considered alongside conventional biochemical markers during the clinical evaluation of patients with HAE-C1INH. Although confirmation in larger multicenter cohorts is required, integrating genotype information into routine assessment may improve prognostic stratification and genetic counseling.

## 5. Conclusions

*SERPING1* variant type was associated with the clinical and biochemical expression of HAE-C1INH. Among symptomatic heterozygous individuals, missense variants were associated with higher residual C1-INH functional activity, later symptom onset, and lower annual attack frequency than pooled non-missense loss-of-function variants. However, the absence of significant differences in other clinical outcomes and severity scores, together with familial clustering and the exploratory nature of the analyses, precludes concluding that missense variants uniformly confer a milder clinical phenotype. Homozygous individuals showed heterogeneous biochemical and clinical profiles across families; therefore, a uniform gene-dosage effect cannot be concluded. These findings are consistent with the current international literature [9,10,11,22] and contribute to the growing evidence on genotype–phenotype associations in populations with high rates of consanguinity. Larger, multicenter studies using family-adjusted analyses and validated severity measures are needed to confirm these associations.

## Figures and Tables

**Table 1:** Family-based distribution of *SERPING1* variants and zygosity among symptomatic individuals with available genetic testing and C1-INH functional results.

Family	Mutation (HGVS)	Mutation Type	Exon	Heterozygotes	Homozygous	ACMG Class	Total Patients
A	p.Glu260 *	nonsense	5	7	–	P	7
B	Large del exon 4	large deletion	4	2	–	P	2
C	Large del exons 5–6	large deletion	5–6	5	–	P	5
D	p.Arg466His	missense	8	1	–	LP	1
E	p.Arg286Alafs*10	frameshift deletion	5	5	–	P	5
F	p.Arg466Cys	missense	8	2	–	LP	2
G	p.Arg400Cys	missense	8	4	3	LP	7
H	p.Met325Thr	missense	6	6	–	LP	6
I	p.Ile379Thr	missense	7	5	2	LP	7
J	p.Ser438Phe	missense	8	3	2	LP	5
K	p.Ser150Phe	missense	3	6	–	LP	6
Total Patients				46	7		53

P, pathogenic; LP, likely pathogenic, * indicates a termination codon. Variant classification according to ACMG/AMP guidelines [16].

**Table 2:** C1-INH functional activity and age at symptom onset according to *SERPING1* variant type among symptomatic heterozygous individuals (*n* = 46).

Mutation Type	*n*	Disease Onset Age (Years), Median (Range)	C1-INH Function (%), Median (Range)
Frameshift deletion	5	6 (5–12)	18.4 (3.4–26.7)
Large deletion	7	7 (4–25)	16.2 (5.8–25.5)
Missense	27	20 (7–70)	28.5 (1–97.7)
Nonsense	7	10 (5–18)	10.9 (1.8–12.4)

C1-INH, C1 inhibitor. Values are presented as median (range).

**Table 3:** Clinical characteristics and disease severity measures according to *SERPING1* variant group among symptomatic heterozygous individuals.

	Missense (*n* = 27)	Pooled Non-Missense LOF (*n* = 19)	*p*
Annual attack frequency (*n*), median (range)	12 (1–227)	48 (4–225)	0.009
Presence of laryngeal attack, *n* (%)	13 (48.1%)	13 (68.4%)	0.232
Hospitalization	6 (22.2%)	2 (10.5%)	0.440
Prophylaxis	14 (51.9%)	7 (36.8%)	0.377
Bygum severity score	7 (2–10) (*n* = 20)	7 (6–8) (*n* = 11)	0.526
Freiberger severity score	7 (3–11) (*n* = 20)	7 (6–10) (*n* = 11)	0.95

C1-INH, C1 inhibitor; HAE, hereditary angioedema.

**Table 4:** Individual clinical, genetic, and biochemical characteristics of the seven homozygous individuals.

Family	Patient ID	SERPING1 Variant	Exon	Age at Symptom Onset, Years	C1-INH Functional Activity, %	HAE Attacks per Year	Laryngeal Attack	HAE-Related Hospitalization	Long-Term Prophylaxis	Bygum Score	Freiberger Score
G	SY61	p.Arg400Cys	8	7	9.9	60	Yes	Yes	Yes	8	9
G	ES66	p.Arg400Cys	8	6	10.5	10	Yes	No	Yes	8	9
G	SD58	p.Arg400Cys	8	6	3.9	2	No	No	Yes	5	6
I	A10	p.Ile379Thr	7	27	18.3	2	No	No	No	NA	NA
I	A20	p.Ile379Thr	7	24	29.6	1	No	No	No	NA	NA
J	D10	p.Ser438Phe	8	4	2.0	48	Yes	Yes	Yes	7	6
J	D20	p.Ser438Phe	8	17	0	12	Yes	No	Yes	NA	NA
Overall				7 (4–27)	9.9 (0–29.6)	10 (1–60)	4/7 57.1%	2/7 28.5%	5/7 71.4%	7.5 (5–8)	7.5 (6–9)

C1-INH, C1 inhibitor; HAE, hereditary angioedema; NA, not available. Bygum and Freiberger scores are reported when available. Data are presented descriptively without formal statistical comparisons.

## Data Availability

The data supporting the findings of this study are available from the corresponding author upon reasonable request. The data are not publicly available because they contain potentially identifiable familial, clinical, and genetic information and are subject to privacy and ethical restrictions.

## Supplementary Materials

The following supporting information can be downloaded at: https://www.mdpi.com/article/10.3390/medicina62091635/s1, Supplementary Figure S1: Pedigrees of the 11 families included in the study; Supplementary Table S1: Individual-level clinical, genetic, and biochemical characteristics of the labelled individuals depicted in the pedigrees.
